# The Inhibition of Mitogen-Activated Protein Kinases (MAPKs) and NF-κB Underlies the Neuroprotective Capacity of a Cinnamon/Curcumin/Turmeric Spice Blend in Aβ-Exposed THP-1 Cells

**DOI:** 10.3390/molecules28247949

**Published:** 2023-12-05

**Authors:** Alessandro Maugeri, Caterina Russo, Giuseppe Tancredi Patanè, Davide Barreca, Giuseppina Mandalari, Michele Navarra

**Affiliations:** 1 Department of Veterinary Sciences, University of Messina, 98168 Messina, Italy; amaugeri@unime.it; 2 Department of Chemical, Biological, Pharmaceutical and Environmental Sciences, University of Messina, 98166 Messina, Italy; carusso@unime.it (C.R.); giuseppe.patane@studenti.unime.it (G.T.P.); davide.barreca@unime.it (D.B.);

**Keywords:** neuroinflammation, Alzheimer’s disease, natural products, curcumin, amyloid, cinnamon, turmeric

## Abstract

Alzheimer’s disease (AD) is a neurodegenerative disorder characterized by an increased level of β-amyloid (Aβ) protein deposition in the brain, yet the exact etiology remains elusive. Nowadays, treatments only target symptoms, thus the search for novel strategies is constantly stimulated, and looking to natural substances from the plant kingdom. The aim of this study was to investigate the neuroprotective effects of a spice blend composed of cinnamon bark and two different turmeric root extracts (CCSB) in Aβ-exposed THP-1 cells as a model of neuroinflammation. In abiotic assays, CCSB demonstrated an antioxidant capacity up to three times stronger than Trolox in the ORAC assay, and it reduced reactive oxygen species (ROS) induced by the amyloid fragment in THP-1 cells by up to 39.7%. Moreover, CCSB lowered the Aβ stimulated secretion of the pro-inflammatory cytokines IL-1β and IL-6 by up to 24.9% and 43.4%, respectively, along with their gene expression by up to 25.2% and 43.1%, respectively. The mechanism involved the mitogen-activated protein kinases ERK, JNK and p38, whose phosphorylation was reduced by up to 51.5%, 73.7%, and 58.2%, respectively. In addition, phosphorylation of p65, one of the five components forming NF-κB, was reduced by up to 86.1%. Our results suggest that CCSB can counteract the neuroinflammatory stimulus induced by Aβ-exposure in THP-1 cells, and therefore can be considered a potential candidate for AD management.

## 1. Introduction

Currently, there are around 55 million individuals living with dementia worldwide, with many cases undiagnosed [1]. Due to the aging population, this number is expected to triple by 2050, increasing the burden of the disease, along with the expense for healthcare [2]. The most prevalent type of dementia is Alzheimer’s disease (AD), which accounts for an alarming 60–80% of all dementia cases [3]. AD is characterized by neuronal loss, due to a pathological buildup of the neurotoxic proteins β-amyloid (Aβ) and hyperphosphorylated tau. Furthermore, it is accompanied by persistent chronic inflammation as shown by activated microglia, which are the immune cells of the central nervous system (CNS), thus playing a significant role in both health and disease [4]. Indeed, an excess of Aβ activates microglia, a process that is further accelerated by the rise in the fibrillar form of this peptide. This innate immune response then contributes to the pathogenesis of AD by raising neurotoxic pro-inflammatory mediators while lowering neuroprotective anti-inflammatory mediators and elevating oxidative stress [5]. For this reason, microglial cells have raised the interest of researchers as a tool to deeply investigate the events occurring during neurodegeneration. In particular, THP-1 cells, human leukemia monocytic cells, stressed with bacterial lipopolysaccharide or Aβ fragments from different origins, have been extensively employed as model for several neurobiological disorders, due to their resemblance to microglial cells [6]. This model can also be used to screen the neuroprotective potentiality of novel candidates, given the ever-growing need of finding new strategies to hamper neurodegeneration. This because, despite AD’s high incidence, the approved medications for its treatment are scarce. Furthermore, none of these medications can halt, reverse, or even just delay the neuronal loss and degeneration that underlies AD symptoms and leads to irreversible pathological changes. Therefore, the scientific community is challenged to better comprehend and develop more efficient strategies to manage this insidious disease [7].

Natural products have gained the attention of researchers seeking novel therapeutic approaches due to their neuroprotective effect, since they are able to target many pathogenic pathways linked to AD [8]. It is noteworthy that galantamine, a cholinesterase inhibitor, is a natural substance, and rivastigmine is a semi-synthetic derivative of the natural molecule physostigmine [9]. Furthermore, it has been suggested that plant mixtures or extracts may have benefits over single substances, owing to their multi-target approaches, which might offer unique therapeutic or preventive strategies for AD given the complexity of its pathophysiology [10]. Recently, natural products have been claimed to protect the blood–brain barrier (BBB), whose damage is another typical pathologic feature of AD [11]. A growing body of research suggests that orally administered herbal formulations may provide certain cognitive benefits to Alzheimer’s disease patients [12]. As a result, numerous phytocompounds from various sources have undergone preclinical and clinical testing to see whether they possess neuroprotective properties that might delay or lessen AD [9]. Some of the most promising results have been obtained using phenolic compounds and polyphenol-rich fractions [9].

On this basis, the aim of the current study was to assess the neuroprotective activity of a cinnamon/curcumin/turmeric spice blend (CCSB), composed of an extract of *Cinnamomum cassia* bark combined with two different root extracts of *Curcumin longa*, in Aβ-exposed THP-1 cells, assessing the molecular pathways underlying its antioxidant and anti-inflammatory effects.

## 2. Results

### 2.1. Chemical Characterization of Cinnamon/Curcumin/Turmeric Spice Blend (CCSB)

**Figure 1:**
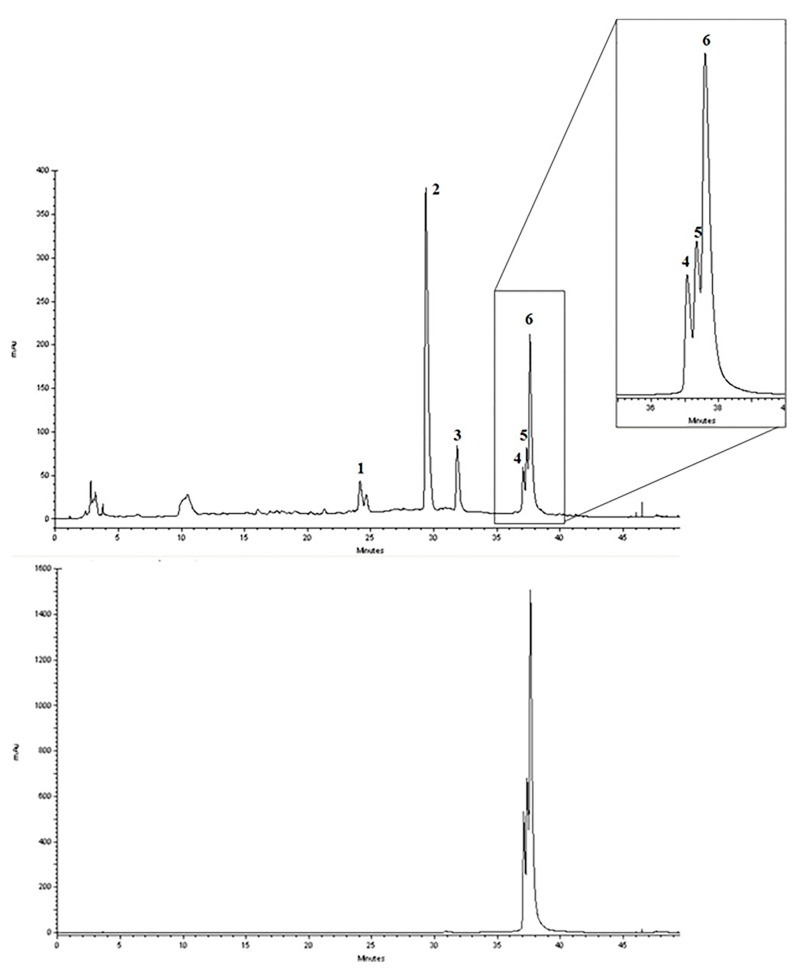
Representative reverse-phase high-performance liquid chromatography coupled with diode array detection (RP-DAD-HPLC) separation of the methanolic extract obtained from CCSB. Coumarin (1); cinnamaldehyde (2); 2-methoxycinnamaldehyde (3); bisdemethoxycurcumin (4); desmethoxycurcumin (5); and curcumin (6). The inset magnified the chromatograms in a 35–40 min interval time. Peak identification was performed by matching retention time and UV spectra against commercially available reference compounds and sample spiking with pure reference compounds.

The quali-quantitative characterization of the methanolic extract of CCSB was recorded at 280 nm and 426 nm, showing its richness in curcuminoids. Taking into consideration the retention time, UV spectra, and sample spiking with pure reference compounds, the main peaks of the chromatogram have been identified as coumarin (1), cinnamaldehyde (2), 2-methoxycinnamaldehyde (3), bisdemethoxycurcumin (4), desmethoxycurcumin (5), and curcumin (6) (Figure 1 and Table 1).

### 2.2. CCSB Possesses Antioxidant Properties

The evaluation of the antioxidant capability of CCSB was performed employing different abiotic models (Table 2). By the Folin–Ciocalteu assay, CCSB demonstrated a phenolic content of 149.2 ± 2.1 mg of gallic acid equivalents (GAE) per gram of dried extract. By the quenching of the stable 2,2-Diphenylpicrylhydrazyl (DPPH) radical and the oxygen radical absorbance capacity (ORAC) assay, we showed that CCSB was able to counteract both peroxyl and nitrogen radicals up to 6787.1 ± 325.1 µmol and 312.1 ± 5.9 mg of Trolox equivalents (TE), respectively. Finally, by potassium ferricyanide reducing antioxidant power (PFRAP) assay, we documented that CCSB could reduce Fe^3+^ ions up to 289.9 ± 9.0 mg of ascorbic acid equivalents (AAE). These data support the valuable antioxidant capability of CCSB.

### 2.3. CCSB Does Not Exert Cytotoxic Effects in THP-1 Cells

**Figure 2:**
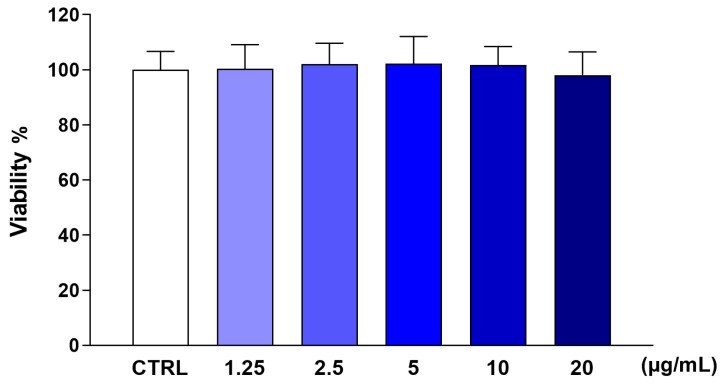
Effect of CCSB on THP-1 cell viability by MTT test. Different concentrations of CCSB (from 1.25 to 20 µg/mL) were added to the culture medium for 24 h, and then cell viability was assessed using the MTT test. Results are expressed as percentages of the values detected in untreated cultures (CTRL). Data are means ± SEM of three independent experiments performed in eight replicates.

Firstly, we assessed the potential toxicity of CCSB in THP-1 monocytes to select the concentrations to be employed in further studies. As shown in Figure 2, the exposure of THP-1 cells to increasing concentrations of CCSB for 24 h did not induce any sign of toxicity, as assessed using the MTT assay, a widely used method to evaluate the mitochondrial activity of living cells and their viability.

**Figure 3:**
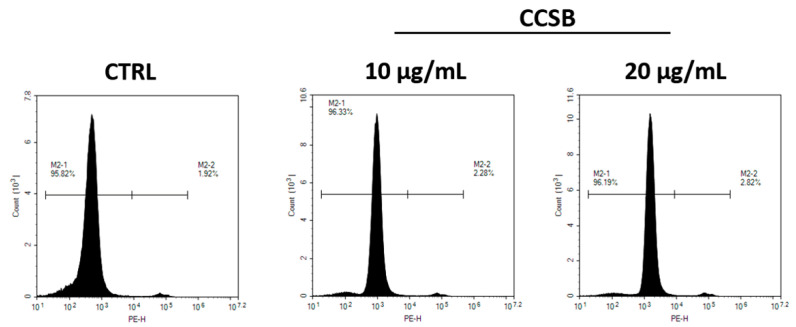
Effect of CCSB on THP-1 cell viability evaluated using PI exclusion assay. Plots display the fluorescence intensity detected in the cells recorded in the FL-3 channel against cell count. Plots are representative of three experiments performed independently.

These data were further confirmed using flow cytometry following the propidium iodide (PI) exclusion assay, which exploits a fluorescent dye that stoichiometrically binds to nucleic acid in damaged cells. In this experiment, we assessed the highest concentration tested in the previous assay (10 and 20 µg/mL) of CCSB, which confirmed no toxicity (Figure 3). Therefore, these two concentrations were further employed in the following experiments.

### 2.4. CCSB Counteracts ROS Production in Aβ-Stressed THP-1 Cells

**Figure 4:**
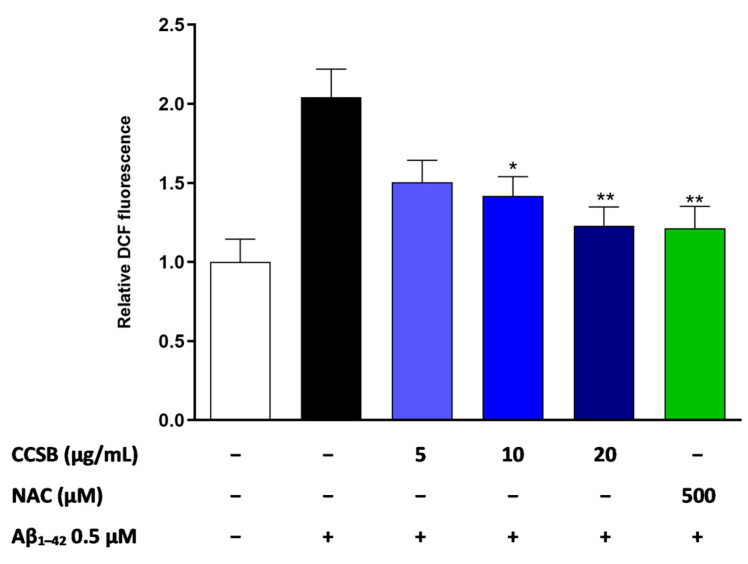
Effect of CCSB on ROS production in Aβ-stressed THP-1. The evaluation of intracellular ROS levels was performed through the fluorometric measurement of oxidized DCF-DA in THP-1 cells incubated with/without 0.5 µM Aβ_1–42_ for 16 h either in the presence or absence of CCSB (5–20 µg/mL) and NAC (500 µM), both added 30 min prior the stressor. Results are expressed as a fold change of DCF-DA fluorescence with respect to untreated cells (white bar) set as 1 and represent the mean ± SEM of three experiments. * *p* < 0.05 and ** *p* < 0.01 vs. Aβ_1–42_-treated THP-1 cells (black bar).

To evaluate the protective effect of CCSB against the oxidative stress caused by the Aβ_1–42_ exposure, the reactive oxygen species (ROS) were quantified employing the 2,7-dichloroflorescein diacetate (DCF-DA) as a probe that quantitatively produces fluorescence when reacting with ROS. As shown in Figure 4, the exposure of THP-1 cells to 0.5 µM Aβ_1–42_ for 16 h (black bar) doubled the intracellular ROS level with respect to untreated cells (white bar). Pre-treatment with increasing concentration of CCSB (from 5 to 20 µg/mL; blue bars) for 30 min was able to hinder the aforementioned outcome in a statistically significant manner for CCSB at 10 and 20 µg/mL by 30.6% (*p* < 0.05) and 39.7% (*p* < 0.01), respectively. Noteworthy, pre-treatment with 0.5 M N-acetylcysteine (NAC), a well-known strong antioxidant, prevented the increase in intracellular ROS with a comparable efficacy as the highest concentration of CCSB (40.5%; *p* < 0.01).

### 2.5. CCSB Hinders the Release of Pro-Inflammatory Cytokines in Aβ-Stressed THP-1 Cells

Since it is known that Aβ_1–42_ exposure in THP-1 cells unleashes a dramatic release of pro-inflammatory cytokines, the next step was to assess the capability of CCSB to counteract this outcome. For this experiment, we employed the concentrations of the blend that possessed significant antioxidant activity, although not inducing any cytotoxic effect (i.e., 10 and 20 µg/mL).

**Figure 5:**
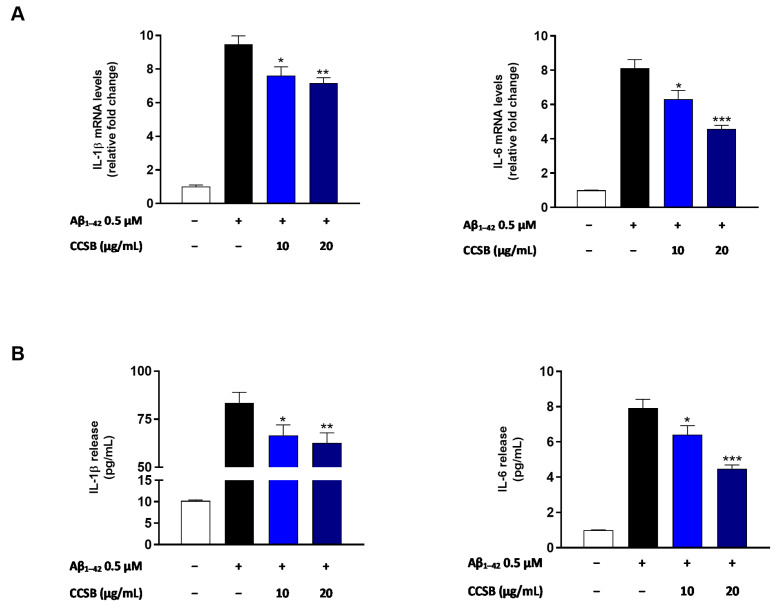
Effect of CCSB on both expression and release of IL-1β and IL-6 mRNA after Aβ_1–42_ exposure of THP-1 cells. (**A**) The quantification of mRNA levels was assessed by real-time PCR and expressed as relative fold change in treated cells compared to the mRNA levels found in untreated culture after normalization to β-actin. (**B**) The quantification of secreted cytokines was assessed by enzyme-linked immunosorbent assay (ELISA) in supernatants of THP-1 monocytes treated and/or untreated with CCSB and Aβ_1−42_. Data are expressed as the mean ± SEM of three experiments performed separately. * *p* < 0.05, ** *p* < 0.01 and *** *p* < 0.001 vs Aβ_1−42_-treated THP-1 cells (black bar).

As shown in Figure 5, the exposure of THP-1 cells to the amyloid fragment caused a strong increase in the mRNA levels of interleukin (IL)-1β and IL-6. Conversely, the pre-treatment with CCSB was able to counteract the injury caused by Aβ_1–42_, significantly decreasing the levels of each cytokine evaluated. Specifically, IL-1β expression was reduced by 20.1% (*p* < 0.05) and 25.2% (*p* < 0.01), while IL-6 expression by 22.1% (*p* < 0.05) and 43.1% (*p* < 0.001) compared t to the Aβ_1–42_ exposure alone for the 10 and 20 µg/mL of CCSB, respectively (Figure 5A). This outcome was also reflected in the release level of these cytokines in the media of THP-1-stressed cells (Figure 5B). Moreover, CCSB, even at the lowest concentration tested (10 µg/mL), significantly decreased the release of both IL-1β and IL-6 (Figure 5B). Indeed, the 10 µg/mL of CCSB decreased the medium concentration of IL-1β and IL-6 by 20.2% (*p* < 0.05) and 18.9% (*p* < 0.05), respectively, compared to Aβ-exposed only cells, and the 20 µg/mL concentration by 24.9% (*p* < 0.01) and 43.4% (*p* < 0.001), respectively.

### 2.6. CCSB Inhibits Mitogen-Activated Protein Kinases (MAPKs) Phosphorylation in Aβ-Stressed THP-1 Cells

**Figure 6:**
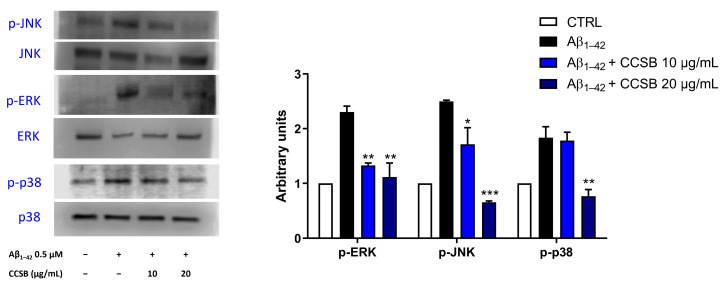
Modulation of MAPKs induced by CCSB in THP-1 cells exposed to Aβ_1–42._ Representative immunoblots of JNK, p38, and ERK 1/2, along with their phosphorylated counterparts, are shown. The blot images were cropped around the region of interest, and samples were resolved on gels run under the same experimental conditions. The relative densitometric analysis of phosphorylated factors is shown, normalized against their total counterpart. These results are expressed as the mean ± SEM of three different experiments. * *p* < 0.05, ** *p* < 0.01 and *** *p* < 0.001 vs. Aβ_1–42_-treated THP-1 cells (black bar).

It is known that the activation of MAPKs cascade plays a role in the Aβ_1–42_ fragment-induced THP-1 cell cytotoxicity. Therefore, the modulation of these proteins using CCSB was evaluated using Western blotting. As expected, the amyloid fragment (0.5 µM for 16 h) increased the phosphorylation of each member of the MAPK superfamily, whereas pre-treatment with CCSB was able to hinder this outcome in a significant manner for ERK and JNK at both concentrations tested (Figure 6). Specifically, CCSB at 10 µg/mL decreased the Aβ-induced ERK phosphorylation by 42.4% (*p* < 0.01) and JNK phosphorylation by 31.3% (*p* < 0.05), whereas CCSB 20 µg/mL reduced ERK phosphorylation by 51.5% (*p* < 0.01) and JNK phosphorylation by 73.7% (*p* < 0.001) compared with Aβ-exposed cells only. In contrast, only the highest concentration of CCSB (20 µg/mL) brought a significant inhibition of Aβ-induced p38 activation, with a reduction of its phosphorylation by 58.2% (*p* < 0.01; Figure 6).

### 2.7. CCSB Counteracts NF-κB Activation in Aβ-Stressed THP-1 Cells

**Figure 7:**
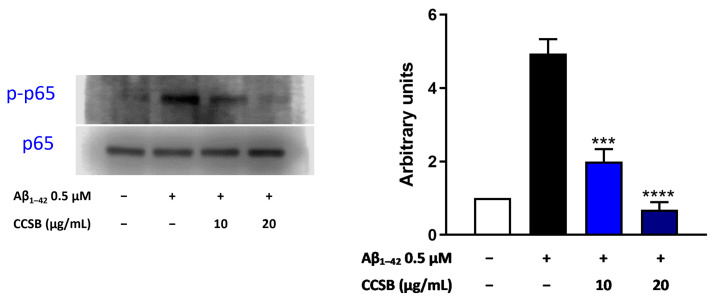
Blockage of NF-κB activation by CCSB in THP-1 cells exposed to Aβ_1–42_. Representative immunoblots of p65, along with their phosphorylated counterpart, are shown. The blot images were cropped around the region of interest, and the samples were resolved on gels run under the same experimental conditions. The relative densitometric analysis of phosphorylated p65, normalized against its total counterpart, is shown. Data are shown as the mean ± SEM of three different experiments. *** *p* < 0.001 and **** *p* < 0.0001 vs. Aβ_1–42_-treated THP-1 cells (black bar).

The most relevant player during inflammation is NF-κB, which is bound to IκBα (NF-κB inhibitor) in the cytoplasm. A neuro-inflammatory trigger, such as that of Aβ_1–42_, releases NF-κB from its inhibitor, which can be phosphorylated and hence translocated into the nucleus to induce the expression of a wide plethora of genes. To this effect, we evaluated the level of phosphorylation of the subunit p65 by Western blotting, which, together with p50, forms NF-κB. Undoubtedly, the amyloid fragment dramatically increased the levels of phospho-p65 (Figure 7, black bar) compared with untreated THP-1 cells. Nonetheless, CCSB hindered this effect, as clearly demonstrated by the lower level of phospho-p65 (Figure 7). Specifically, CCSB at 10 µg/mL reduced the phosphorylation of p65 by 59.5% (*p* < 0.001) and at 20 µg/mL by 86.1% (*p* < 0.0001) compared with THP-1 cells exposed to the amyloid fragment only (Figure 7).

## 3. Discussion

In this study, for the first time, we showed that CCSB possessed antioxidant capacity both in cell-free and in THP-1 cells stressed with Aβ_1–42_. Moreover, we showed that CCSB was able to counteract the pro-inflammatory effect of the amyloid fragment in THP-1 cells via the inhibition of both MAPKs and NF-kB pathways, thus suggesting its potential employment as a natural product to hamper neurodegeneration. On this line, the plant kingdom offers an extremely vast number of phytochemicals potentially able to prevent or delay the degeneration of brain functions [13]. This can be achieved by plant secondary metabolites able to influence the pro-inflammatory and pro-oxidant status, typical of neurodegeneration [14]. In this field, the rhizome of the plant *Curcuma longa*, commonly known as turmeric, has gathered great attention for its beneficial properties. It is mainly composed of curcuminoids (i.e., curcumin, desmethoxycurcumin, bisdemethoxycurcumin), which are responsible for the strong yellow hue of this spice. Researchers are particularly interested in curcumin because it possesses a wide range of effects, including anti-inflammatory, antioxidant, and anti-AD properties [15]. Curcuminoids are claimed to limit the development of Aβ oligomers, and tau aggregation, as well as to have anti-inflammatory and antioxidant activities [16]. Among other plants investigated for their neuroprotective properties, *Cinnamomum cassia* has been researched [17]. It is one type of cinnamon used as a food spice obtained from the inner bark of the Lauraceae family of evergreen trees of the genus *Cinnamomum*. Its main bioactive compound is cinnamaldehyde, representing almost 90% of the whole cinnamon essential oil, followed by coumarins and other oxygenated compounds [18].

In this study, we focused on a spice blend composed of a combination of cinnamon, *Cinnamomum cassia* (bark) and two different root extracts of turmeric, *Curcuma longa*. This blend was originally formulated to be employed in a dietary supplement, as part of a holistic approach to whole body glucose management [19]. The effects of this supplement have been studied in a series of pilot human trials in which the acute and chronic effects were investigated. Alongside certain metabolic effects, there were indications of improvements in aspects of cognitive performance in individuals [19]. This is consistent with other reports of cognitive benefits of cinnamon and curcumin/turmeric [20,21]. Indeed, cinnamon and its main constituents (i.e., cinnamaldehyde and cinnamic acid) were shown to improve memory and learning by lowering amyloid plaque in the hippocampus and tau-protein phosphorylation, thanks to their antioxidant, anti-inflammatory, and anti-cholinesterase action, along with neurotrophic impact, neural maintenance, and insulin signaling enhancement [22]. Indeed, in vitro investigations demonstrated considerable favorable outcomes when cinnamon and cinnamaldehyde were used to reduce neuronal mortality and Aβ-buildup; in vivo investigation, verified the considerable beneficial effect in the two principle categories of cognitive function (i.e., memory and learning) using behavioral tests [22]. Regarding turmeric and, particularly, its main constituent curcumin, it has been reported, through pre-clinical investigations, that it was able to correct or prevent disease-induced cognitive decline rather than improve further ‘normal’ cognitive performance. This is most likely due to curcumin’s capacity to act directly on Aβ plaques and to its anti-inflammatory and antioxidant capabilities. Confirming the pre-clinical findings, some clinical studies also indicate protective effects of curcumin against cognitive decline, although the weight of evidence should clearly be strengthened [23].

In our present exploratory study, we first assessed the chemical composition of CCSB by means of RP-DAD-HPLC, a methodic which allows a precise and thorough evaluation of complex matrices as natural extracts. With CCSB being composed of two different root extracts of turmeric, our results showed a prevalence of curcuminoids in its composition. In detail, we showed that almost the 90% of the whole curcuminoids are represented by curcumin. This is in line with previous reports which claim that turmeric extracts contain this particular percentage of curcumin [24]. Interestingly, it has also been reported that the common amount of curcumin in dry turmeric extracts is about 3–5%, yet here we found that CCSB gathers more than three times this amount, potentially due to the presence of oleoresin in its composition. Regarding cinnamaldehyde, 2-methoxycinnamaldehyde, and coumarin, these represent the main component of cinnamon bark essential oil. The prevalence of cinnamaldehyde over the other two components is in line with the current literature [25].

Following qualitative and quantitative analyses, we focused on the abiotic evaluation of CCSB antioxidant activity. We showed that CCSB was able to hamper oxygen radical formation in the ORAC assay in a stronger manner than Trolox, used as standard, in contrast to DPPH assay. This can be explained by the fact that the DPPH radical possesses a greater steric effect respect the smaller ROO^•^ of the ORAC assay [26]. Nevertheless, our results confirm the antioxidant effect of CCSB in cell free setting. In addition, we showed that CCSB was able to reduce both Folin–Ciocalteu reagent and potassium ferricyanide, as suggested by previous reports [27].

In this study, we employed human THP-1 monocytes exposed to Aβ, an in vitro model exploited to mimic AD and assess the neuroprotective activity of natural products [28,29]. This is because these cells resemble the microglia of the human brain, which are known to unleash the inflammatory machinery during AD development [30]. Notably, it has been demonstrated that THP-1 monocytes can be activated by fibrillar Aβ_1−42_, bringing an increase in the production of several pro-inflammatory cytokines [31]. These findings are significant since it has been described that peripheral hemopoietic cells (e.g., monocytes) are able to cross the BBB and differentiate into microglial cells within the brain, indicating that they arise from peripheral hemopoietic cells [32]. Furthermore, AD is known to damage BBB thus altering the transportation of substances, both harmful and beneficial to the brain. Indeed, the hampered BBB does not allow Aβ to pass from the brain parenchyma to the peripheral circulation, thus causing neuroinflammation and oxidative stress [33].

Oxidative stress acts as a link between the many processes underlying AD [34]. It damages neurons and occurs through a variety of pathways. Accordingly, previous research has shown that Aβ can stimulate the increase of ROS and induce oxidative stress [35]. In this study, we observed an increase in ROS in THP-1 cells exposed to the Aβ fragments, an event that was hindered by the addition of CCSB, to a magnitude comparable to NAC, a well-known and powerful antioxidant. This agrees with previous research which has shown that both cinnamon and turmeric possess radical scavenging properties, such as those we demonstrated here, in several in vitro and in vivo models [36]. In more detail, it has been suggested that both curcumin and cinnamaldehyde, the two main components of turmeric and cinnamon, respectively, can contribute to the depletion of intracellular ROS via their chelating activity. This interrupts the redox reactions exacerbated in damaged neuron cells, thus lowering the oxidative stress [36].

Another hallmark of neurodegeneration is inflammation, whose main drivers are cytokines. Indeed, altered levels of these messengers or other inflammatory markers correlate with the severity of AD [37]. More specifically, IL-6 and IL-1β have been reported to be increased during AD [38]. Interestingly, our results demonstrated that CCSB reduced both the release and gene expression of these cytokines, thus supporting the beneficial properties of the blend. This is in line with other reports in which cinnamon and turmeric extract showed anti-inflammatory potential against neurodegeneration [39]. Indeed, curcuminoids are said to be the most potent cytokine-suppressive anti-inflammatory drugs and have a much broader anti-inflammatory action respect to the conventional non-steroidal anti-inflammatory drugs [40]. Similarly, cinnamon extracts are able to regulate the over-production of cytokines in chronic inflammatory status, which may lead to organ degeneration and failure, such as iatrogenic colitis [41].

MAPKs are important signal transducers that have developed to convey a diverse range of extracellular signals to intracellular signaling cascades [42]. From cell proliferation and differentiation to cell survival, these cascades govern practically every function of a mammalian cell. In the pathology of AD, numerous researchers have claimed that MAPK are important due to their multifactorial potential [43]. As expected, the Aβ fragment stimulated the phosphorylation of ERK 1/2, JNK, and p38 MAPKs, as a result of the strong pro-oxidant and pro-inflammatory stimulus arisen, which was impeded by CCSB. Our data agrees with those of Kim and collaborators [44], who showed that cinnamaldehyde produced anti-inflammatory effects in RAW 264.7 murine macrophages by inhibiting ERK, JNK and p38 MAPKs phosphorylation. Moreover, bisdemethoxycurcumin was reported to diminish the inflammatory status in a food allergy murine model sensitized after oral challenge with ovalbumin via the inhibition of MAPKs phosphorylation [45].

The NF-κB family is made up of five transcription factors, which are involved in numerous physiological processes, along with mediating inflammatory responses [46]. In microglial cells, the activation of NF-κB signaling, followed by the release of cytokines and chemokines, culminates in the persistent inflammation seen in AD [47]. In this study, we observed that CCSB was able to reduce the phosphorylation of p65 triggered by the Aβ fragment in THP-1 cells, hence its translocation into the nucleus to promote the transcription of inflammatory-linked genes. In this respect, it was reported that a polyphenolic cinnamon fraction exhibited anti-inflammatory properties in a monocyte/macrophage model, via the inhibition of the NF-κB pathway and, hence, cytokine release [48]. Similarly, curcumin has been shown to suppress the pro-inflammatory stimulus elicited by bacterial endotoxin in THP-1 cells blocking the NF-κB activation [49]. 

Interestingly, it has been recently reported that neurodegeneration induced by Aβ deposition in the brain can mitigated physiologically by the cerebrospinal fluid (CSF) flow, which is able to drain out this toxic fragment. Nevertheless, an increase of the inflammatory status due to an abnormal presence of Aβ fragments is thought to alter the normal production, turnover, and resistance of CSF, thus starting a vicious cycle that worsens the pathology. In particular, if CSF production failure predominates, AD develops, while if resistance to CSF outflow predominates, normal-pressure hydrocephalus results, a condition characterized by an abnormal deposition of CSF in the brain’s ventricles. The disorders may eventually converge in vulnerable individuals, resulting in a hybrid entity called NPH-AD [50]. Notably, there are chirurgical strategies to lessen the NPH and, hence, increase the outflow of CSF to increase the drainage of Aβ fragments [51]; yet, given the inflammatory onset of this phenomenon [52], the employment of anti-inflammatory natural products as CCSB may play a crucial role in the fight against neurodegeneration on multiple fronts.

## 4. Materials and Methods

### 4.1. Chemical Characterization

#### 4.1.1. Reagents and Standard Solutions

HPLC-grade acetonitrile and methanol were from Sigma-Aldrich (St. Louis, MO, USA), and coumarin, cinnamaldehyde, 2-methoxycinnamaldehyde, bisdemethoxycurcumin, desmethoxycurcumin and curcumin were supplied from Glentham Life Sciences Ltd. (Corsham, Wiltshire, UK) and Sigma-Aldrich (St. Louis, MO, USA). The other chemicals and reagents utilized in this investigation were all analytical grade and were acquired from Sigma (St. Louis, MO, USA).

#### 4.1.2. Preparation of Methanol Extract

The powder of the CCSB (1.0 g) was extracted at room temperature under continuous stirring for 2 h with methanol (1:200 *w*/*v*). Afterwards, samples were centrifuged (2500 rpm) for 10 min, and the supernatants were filtered with filter paper and collected in a balloon. This procedure was repeated n times until we reached an exhaustive extraction of the compounds present in the power. The collected fractions were evaporated in a rotavapor until we reached a ratio with the starting fresh leaves material of 1:300. The samples were stored at −20 °C in the dark until further analysis. Each sample was filtered through an Iso-Disc P-34, 3 mm diameter PTFE membrane, and 0.45 µm pore size (Supelco, Bellefonte, PA, USA) prior to RP-HPLC-DAD separation.

#### 4.1.3. RP-DAD-HPLC Separation and Identification

Reverse phase-diode array detector-high performance liquid chromatography (RP-DAD-HPLC) was performed by a Shimadzu system (Shimadzu Ltd., Canby, Oregon, USA). The separation of each compound was carried out on a 250 mm: 4.6 mm i.d., 5 mm Discovery C18 column (Supelco, Bellefonte, PA), equipped with a 20 mm: 4.0 mm guard column, which was then put in an oven set at 30 °C. The injection loop was 20 µL, employing a flowrate of 1.0 mL/min. The mobile phase involved a linear gradient of acetonitrile in H_2_O as follows: 5–20% (0–15 min), 20–30% (15–20 min), 30–50% (20–30 min), 50–100% (30–35 min), 100% (35–40 min), 100–5% (40–50 min) and 5% (50–60 min). The UV spectra were recorded from 200 nm to 600 nm, and diode array simultaneous detection was carried out at 254, 278, 320, 350, 425 and 520 nm. Each sample was assessed three times, and produced superimposable chromatograms. Peak identification was carried out by matching retention time and UV spectra respect to commercially available reference compounds and spiking the samples with pure reference compounds.

#### 4.1.4. Standard Solutions

Stock solutions of coumarin, cinnamaldehyde, 2-methoxycinnamaldehyde, bisdemethoxycurcumin, desmethoxycurcumin and curcumin (1 mg/mL) were made in HPLC grade methanol and stored at 4 °C in dark vials, with a stability of 1 month. The working standard solutions were prepared each day by diluting stock solutions with HPLC grade methanol. Vicenin-2 was used as external standard.

#### 4.1.5. Calibration and Linearity

By directly injecting a range of standard concentrations into the HPLC system, the linearity of the method was assessed. The elution was performed as previously mentioned, and by graphing the concentration of standard compounds against peak areas (average of three runs), standard calibration curves were created. To represent typical flavonoid concentrations in the natural matrices used to prepare the blend, the calibration range was selected. Plotting the ratios of compound peak areas to those of the external standard against known concentrations of pure compounds allowed for the construction of calibration curves, as well as the extraction of linear regression equations. The percentage of the ratio between the peak areas of the compounds in a processed spiked sample standard and the same compounds in a pure standard solution was used to compute the extraction recovery. By means of S/N = 3, the lowest analyte concentration detectable above the system noise level was identified as the limit of detection (LOD). The areas of each chromatogram peak were integrated at 425 nm for curcumin, desmethoxycurcumin, and bisdemethoxycurcumin, and at 280 nm for coumarin, cinnamaldehyde, and 2-methoxycinnamaldehyde. The lowest standard concentration (LOQ) is the lowest concentration that can be found with a precision and accuracy of less than 20%.

### 4.2. Cell-Free Antioxidant Capacity Evaluation

#### 4.2.1. Folin–Ciocalteu Method

The total phenolic content of CCSB was determined using the Folin–Ciocalteu-assay, as described by Kubica et al. [53]. Briefly, 50 μL of methanol/water solutions of different sample concentrations were added to 450 μL of deionized water, 500 μL of a Folin–Ciocalteu reagent, and 500 μL of a 10% sodium carbonate solution and incubated in the dark at room temperature for 1 h, vortexing every 10 min. Absorbance was recorded at 786 nm (Prixma UV-Vis Spectrophotometers) against a blank. Total phenol content was expressed in mg of gallic acid equivalents (GAE/g of CCSB).

#### 4.2.2. Quenching of the Stable 2,2-Diphenylpicrylhydrazyl (DPPH) Radical

The DPPH assay was employed to evaluate the radical scavenging activity of CCSB, as described by Russo et al. [54]. Different concentrations of methanol/water solution of CCSB or vehicle alone (37.5 μL) were added to 1.5 mL of the DPPH methanolic solution (25 mg/L). The absorbance was recorded at 517 nm 30 min after starting the reaction, and the free radical scavenging capacity is expressed in mg of Trolox equivalents per gram of CCSB (TE/g of CCSB).

#### 4.2.3. Oxygen Radical Absorbance Capacity (ORAC) Assay

The radical scavenging activity of CCSB against peroxyl radicals arising from 2,2′-azobis(2-amidinopropane) dihydrochloride (AAPH) was assessed by the ORAC method, as described by Cirmi et al. [55]. Briefly, several concentrations of CCSB (20 μL), prepared in 75 mM phosphate buffer solution (pH 7.4), were mixed with a solution of fluorescein 417 nM (120 µL) and incubated at 37 °C for 15 min. Afterwards, 60 μL of AAPH 40 mM was added. The resulting quenching of fluorescence was recorded every 30 sec for 90 min (excitation: 485 nm; emission: 520 nm; FLUOstar Omega, BMG Labtech, Gainesville, FL, USA). The blank, using phosphate buffer, and calibration solutions of Trolox (10–100 μM) were also included. The ORAC value was calculated employing the area under the curves of fluorescence decay and is expressed in μmoles of TE/g of CCSB.

#### 4.2.4. Reducing Power

The reducing power of CCSB was determined as described by Sharma et al. [56]. In detail, 0.2 mL of several concentrations of CCSB were mixed with 0.5 mL of 0.2 M sodium phosphate buffer (pH 6.6) and 0.5 mL of 1% K_3_Fe(CN)_6_. These solutions were incubated in a water bath at 50 °C for 20 min. Subsequently, 0.5 mL of 10% TCA was added to the mixture, which was then centrifuged at 8300× *g* for 10 min. Afterwards, the supernatants (0.5 mL) were mixed with 0.5 mL of distilled water and 0.1 mL of 0.1% ferric chloride solution. The absorbance was recorded at 700 nm. Reducing power is expressed in mg of ascorbic acid equivalent (AAE) per gram of CCSB.

### 4.3. Cell (In Vitro) Studies

#### 4.3.1. Cell Culture

The human leukemia monocytic THP-1 was originally obtained from ATCC (Rockville, MD, USA) and cultured in RPMI 1640 medium with 10% (*v*/*v*) heat-inactivated fetal bovine serum (FBS), L-glutamine (2 mM), HEPES (10 mM), sodium pyruvate (1 mM), glucose (2.5 g/L), 2-mercaptoethanol (0.05 mM), penicillin (100 IU/mL) and streptomycin (100 µg/mL), at 37 °C in a 5% CO_2_ air humified atmosphere. Each reagent for cell handling was from Gibco (Life Technologies, Monza, Italy).

#### 4.3.2. Cytotoxicity Evaluation of CCSB

The assessment of the potential cytotoxicity of CCSB was performed by both 3-(4,5-dimethylthiazole-2-yl)-2,5-diphenyltetrazolium bromide (MTT) test [57] and propidium iodide (PI) exclusion assay [58]. 

For the MTT test, THP-1 cells were seeded in 96-well plates (5 × 10^4^ cells/well) and treated, after 24 h, with increasing concentrations of CCSB (1.25–20 µg/mL). After further 24 h, treatments were substituted with phenol red-free fresh medium containing 0.5 mg/mL of MTT (Sigma-Aldrich, Milan, Italy), and plates were kept at 37 °C for 4 h. Then, the formazan crystals were dissolved in 100 µL of a 0.1 N HCl/isopropanol lysis solution. The absorbance was measured at a wavelength of 570 nm (reference at 690 nm), using a microplate reader (Bio-Rad Laboratories, Milan, Italy). Results were expressed as cell viability percentage compared to untreated cells, which were arbitrarily set as 100%. Each experiment was performed in eight replicates and repeated three times.

For the PI exclusion assay, THP-1 cells were seeded in 24 well-plate (5 × 10^5^ cells/well) and treated with CCSB (1.25–20 µg/mL) for 24 h. Then, cells were collected, washed and resuspended in 400 µL PBS. Afterwards, cells were incubated with 10 µL of PI labeling solution (10 µg/mL; Sigma-Aldrich) at room temperature for 30 min in the dark. Dead cells were analyzed by Novocyte 2000 cytofluorimeter (ACEA Biosciences Inc., San Diego, CA, USA) with FL-2 channel (10,000 events minimum). The percentage of dead cells was calculated versus non-treated cells.

#### 4.3.3. Determination of the Reactive Oxygen Species (ROS)

The production of ROS was measured as previously described [59]. THP-1 cells were seeded in 96-well plates (5 × 10^4^ cells/well) and incubated with 0.5 µM Aβ_1−42_ for 16h in presence or absence of CCSB (5–20 µg/mL) and NAC (500 µM), added 30 min prior the amyloid fragment. ROS were quantified employing the probe 2′,7′-dichlorodihydrofluorescein diacetate (DCFH-DA) at 25 µM (Sigma-Aldrich). The fluorescence was recorded by a microplate reader (POLARstar Omega, BMG Labtech, Ortenberg, Germany) at 485 nm excitation and 535 nm emission.

#### 4.3.4. Evaluation of Cytokine Secretion and Expression

For the mRNA levels assessment of cytokines, THP-1 cells were plated in Petri dishes (5 × 10^5^ cells/mL) and treated as described above for ROS determination. Then, cells were harvested by centrifugation, and total RNA was isolated using TRIzol (Invitrogen, Carlsbad, CA, USA), as shown [28]. An equal quantity of total RNA (2 µg) for each sample was reverse transcribed into cDNA using the High-Capacity cDNA Archive Kit (Applied Biosystems, Life Technologies, Foster City, CA, USA). Afterwards, quantitative PCR reaction (qPCR) was performed using a 7500 qPCR System (Applied Biosystems), in a total volume of 20 µL, including 1x SYBR® Select Master Mix (Applied Biosystems), 0.2 µM of specific primers and 25 ng of cDNA. Data were analyzed via the 2^−∆∆CT^ relative quantification method versus β-actin (ACTB), used as endogenous control. The values are shown as n-fold change with respect to untreated cells.

To detect human chemokine secretion, an enzyme-linked immunosorbent assay (ELISA) was carried out in cell supernatants, which were recovered, and ten-fold concentrated by freeze-drying. Briefly, according to the manufacturer’s procedure, 50 μL of standards or samples were incubated in 96-well plates at room temperature for 3 h under constant shaking. After washing, 100 μL of the provided substrate solution were added to each well and the plates were kept in the dark for 10 min. The reaction was stopped, and the absorbance was recorded at 450 nm using a microplate reader (Bio-Rad Laboratories). All experiments were performed in triplicate.

#### 4.3.5. Protein Expression Studies

For the evaluation of protein expression, we followed the already described procedures [60]. THP-1 cells were seeded in 100 mm Petri dishes (1 × 10^6^ cells/dish) and treated as explained above. Cells were then harvested, washed with PBS, and lysed using RIPA buffer (Sigma-Aldrich), plus 1% cocktail protease and phosphatase inhibitors (Sigma-Aldrich). The lysates were centrifuged at 12,000× *g* for 15 min at 4 °C and supernatants were collected. Protein quantification was determined employing Bio-Rad DC Protein Assay (Bio-Rad Laboratory). Equal amounts of proteins (30 μg/lane) were separated by 10% sodium dodecyl sulphate-polyacrylamide gel electrophoresis (SDS-PAGE) and electro-transferred on a polyvinylidene fluoride (PVDF) or nitrocellulose membranes (GVS Life Sciences, Sanford, ME, USA). Non-specific binding sites were blocked with 5% (*w*/*v*) non-fat dry milk. Then, membranes were incubated overnight at 4 °C with primary antibodies for total and phosphorylated JNK, ERK1/2, p38 and p65, all diluted 1:1000 in milk and purchased from Cell Signaling Technology (Danvers, MA, USA). Membranes were then washed three times in Tris-Buffered Saline containing 0.15% of Tween 20 (TBS-T) and incubated with horseradish peroxidase-conjugated goat anti-rabbit IgG secondary antibodies (1:5000, Sigma-Aldrich) for 2 h at room temperature. The chemiluminescent signals of protein bands were recorded by C-Digit Blot Scanner (Li-COR Bioscience, Lincoln, NE, USA) with Luminata Forte Western HRP Substrate (Millipore, MA, USA) as substrate. Protein bands were quantified employing Image Studio 5.5.4 software (Li-COR Bioscience).

#### 4.3.6. Statistical Analysis

The data are expressed as mean ± standard error of the means (SEM) and were statistically evaluated for differences using one-way analysis of variance (ANOVA), followed by the Dunnett’s multiple comparison test (GraphPad Prism 8 Software for Science, San Diego, CA, USA). P-values less than or equal to 0.05 were considered significant.

## 5. Conclusions

**Figure 8:**
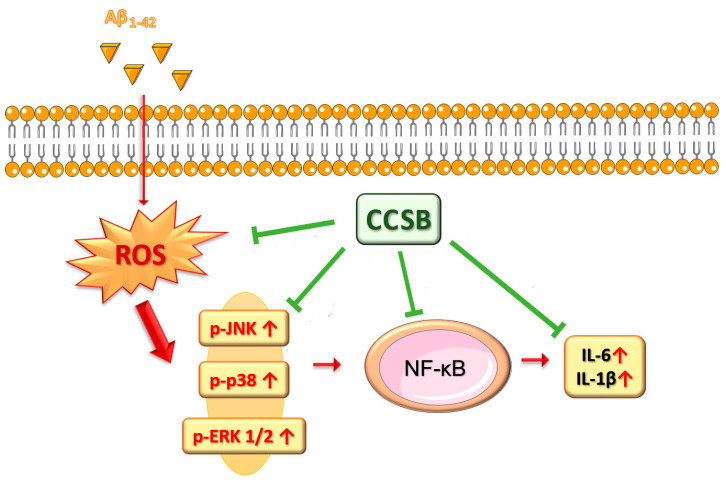
Pathways modulated by CCSB in THP-1 stressed with Aβ_1–42_.

Our results in this experimental cell model of early-stage AD suggest that a spice blend composed of cinnamon, curcumin, and turmeric (CCSB) can significantly alleviate the damage induced by the Aβ fragment in THP-1 cells via the reduction of ROS, hindering both MAPKs and NF-κB activation, and subsequently decreasing pro-inflammatory cytokine levels (Figure 8). Therefore, the combination of cinnamon/curcumin/turmeric in CCSB in dietary supplements, nutraceuticals or functional foods may be considered within the overall strategy for the management of neurodegenerative diseases, such as AD. 

## Figures and Tables

**Table 1:** Quantitative determination of the identified compounds in the CCSB and their structures.

		mg/g	
		Mean	SD
Coumarin	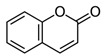	0.018	0.010
Cinnamaldehyde	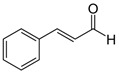	0.125	0.023
2-Methoxycinnamaldehyde	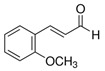	0.037	0.011
Bisdemethoxycurcumin	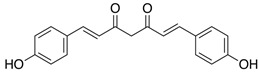	8.763	1.058
Desmethoxycurcumin	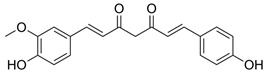	19.156	1.930
Curcumin	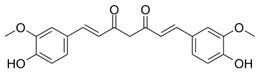	173.022	20.091

**Table 2:** Antioxidant activity of CCSB was evaluated using cell-free assays. Results are reported as mean ± SEM of three independent experiments performed in triplicate and expressed in a standard equivalent/g of dried extract.

ORAC (µmol TE/g)	6787.1 ± 325.1
DPPH (mg TE/g)	312.1 ± 5.9
Folin–Ciocalteu (mg GAE/g)	149.2 ± 2.1
Reducing Power (mg AAE/g)	289.9 ± 9.0

## Data Availability

Data are contained within the article.
